# Early queen infection shapes developmental dynamics and induces long‐term disease protection in incipient ant colonies

**DOI:** 10.1111/ele.13907

**Published:** 2021-11-01

**Authors:** Barbara Casillas‐Pérez, Christopher D. Pull, Filip Naiser, Elisabeth Naderlinger, Jiri Matas, Sylvia Cremer

**Affiliations:** ^1^ IST Austria (Institute of Science and Technology Austria) Klosterneuburg Austria; ^2^ Department of Cybernetics Faculty of Electrical Engineering Czech Technical University in Prague Czech Republic; ^3^ Present address: Department of Zoology University of Oxford Oxford United Kingdom; ^4^ Present address: Department of Chemistry and Pharmaceutical Sciences Vrije Universiteit Amsterdam The Netherlands

**Keywords:** automated behavioural monitoring, colony ontogeny, host–pathogen interactions, life‐history trade‐offs, social insects, transgenerational immunity (TGI)

## Abstract

Infections early in life can have enduring effects on an organism's development and immunity. In this study, we show that this equally applies to developing ‘superorganisms’––incipient social insect colonies. When we exposed newly mated *Lasius niger* ant queens to a low pathogen dose, their colonies grew more slowly than controls before winter, but reached similar sizes afterwards. Independent of exposure, queen hibernation survival improved when the ratio of pupae to workers was small. Queens that reared fewer pupae before worker emergence exhibited lower pathogen levels, indicating that high brood rearing efforts interfere with the ability of the queen's immune system to suppress pathogen proliferation. Early‐life queen pathogen exposure also improved the immunocompetence of her worker offspring, as demonstrated by challenging the workers to the same pathogen a year later. Transgenerational transfer of the queen's pathogen experience to her workforce can hence durably reduce the disease susceptibility of the whole superorganism.

## Graphical abstract

We determined the long‐term impact of early disease stressors on the development and immunity of social insect colonies, after exposing colony‐founding ant queens to low levels of a fungal pathogen. While queen pathogen exposure transiently impaired colony growth, it equipped the workforce with a long‐term immunisation against later challenge to the same pathogen.

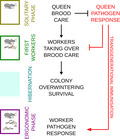

## INTRODUCTION

Stress experienced early in life can have long‐lasting ramifications that impact an organism's developmental trajectory and future fitness. For example, nutritional, temperature and disease stressors can affect traits such as growth, reproduction, behaviour and immunity (McNamara & Buchanan, 2005). The enduring impact of early disease stressors is particularly complex and challenging to predict as they can have direct and indirect effects on hosts. For example, chronic infections may be suppressed by the immune system under favourable conditions, and so be relatively benign, yet can still cause disease if the host becomes otherwise stressed (Kennedy, 2020). Conversely, even if a pathogen is cleared by the host, mounting an immune response itself is costly, often involving the reallocation of resources away from growth and reproduction (Rauw, 2012; Schwenke et al., 2017; Sheldon & Verhulst, 1996). Early‐in‐life infections can also induce life‐long immunological protection, which can even be transferred to the offspring through transgenerational effects (Burton & Metcalfe, 2014; Roth et al., 2018).

The diverse consequences of early‐in‐life infection are well characterised in humans and other vertebrates (e.g. Robertson et al., 2019) but studies on invertebrates typically span much shorter periods (Kurtz & Milutinović, 2016), from a few weeks in fruit flies (Mondotte et al., 2020) to several months in oysters (Green & Speck, 2018). Yet, many invertebrates are much longer lived and, similar to vertebrates, some even pass through extended periods of development before reaching sexual maturity. For example, *Lasius niger* ant queens live several decades and their workers up to 3 years (Kramer et al., 2016), providing that colonies survive the founding stage and subsequent ergonomic growth phase. Those that do will reach reproductive maturity after several years, and produce new sexual offspring (Bourke & Franks, 1995). The protracted development of a colony from a single germline‐like queen giving rise to physically distinct and sterile soma‐like workers is analogous to the ontogeny of multicellular plants and animals, leading to the concept of the superorganism (Boomsma & Gawne, 2018; Wheeler, 1911). As in multicellular organisms, early pathogenic stressors may have enduring consequences on the growth and development of superorganisms. Moreover, trade‐offs between growth and immunity are also expected to be affected by other biotic and abiotic factors, such as seasonal environmental change. Despite the wealth of data on short‐term pathogenic effects and collective disease defences (Cremer et al., 2018), we currently understand little about the long‐term impacts of disease on superorganisms, which are particularly relevant given the ecological and economic importance of social insects and the long lifespan their colonies can reach.

To test how early pathogen exposure of the queen influences the development and long‐term immunity of their incipient colonies, we exposed *L*. *niger* queens directly after their mating flight to low doses of the fungal pathogen *Metarhizium brunneum*, which queens naturally can encounter during nest founding (see methods and Cremer et al., 2018; Pull & Cremer, 2017). For 1 year, we tracked colony development and implemented image analysis‐based automated monitoring of colony behaviours. Additionally, we quantified queen pathogen load throughout and tested the susceptibility of workers from 1‐year‐old colonies to the same pathogen their mother experienced. Monitoring how low‐level queen exposure after flight impacted colony growth, hibernation success, queen infection progression and long‐term transgenerational immunity effects, allowed us to study the effects of early pathogen stress for superorganism ontogeny in the social Hymenoptera, where––contrasting to termites––no king is present that could buffer the negative effects of queen infection (Cole et al., 2018; Cole & Rosengaus, 2019).

## METHODS

**FIGURE 1:**
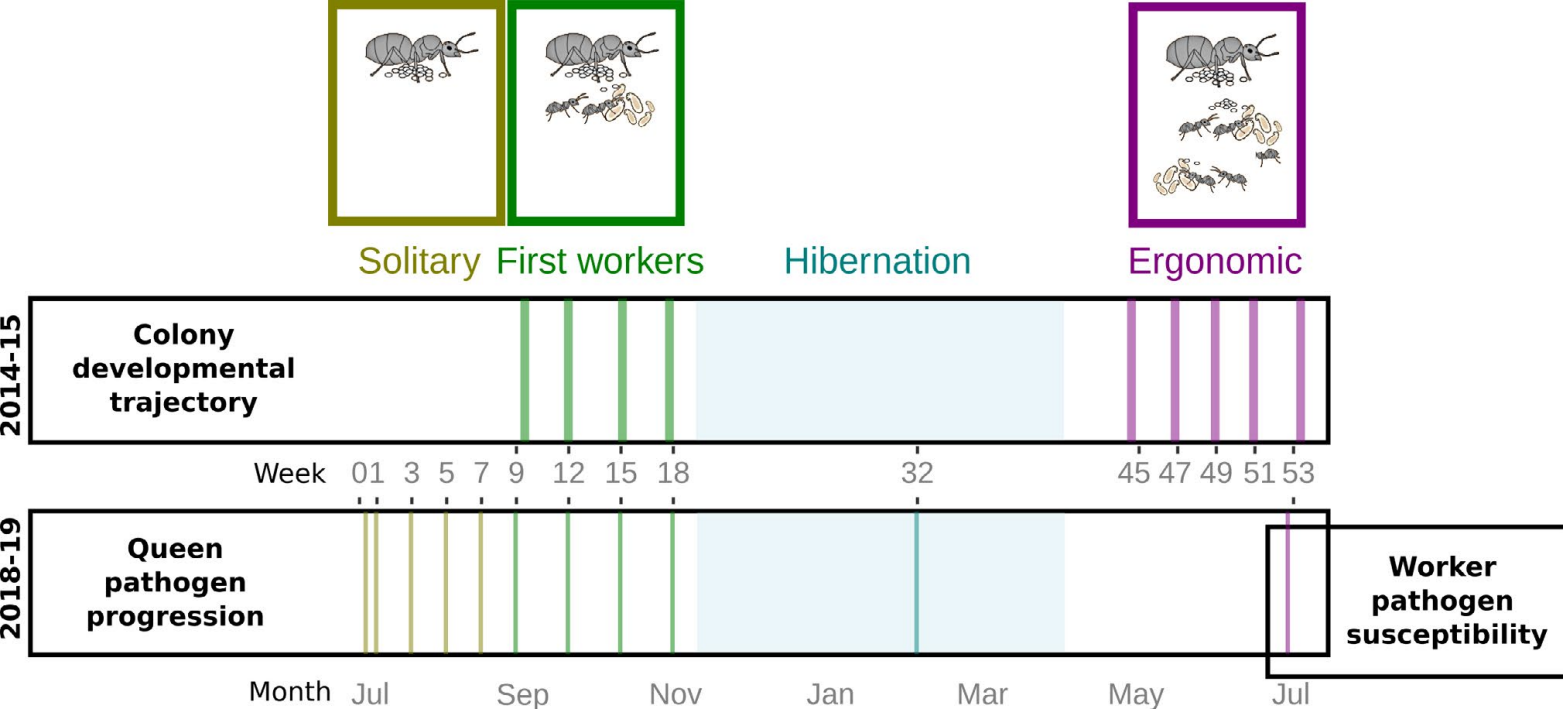
Experimental design. We studied colonies founded by field collected and then experimentally pathogen‐ or sham‐treated queens following their natural mating flights in 2014, resp. 2018, each over the course of 1 year. In the experiment set up in 2014, we analysed colony development (production of brood and workers, hibernation survival), as well as colony behaviour (locomotion, foraging and aggregation around the queen) in video filming sessions before (green) and after (purple) hibernation, corresponding to the first worker phase and the ergonomic growth phase, respectively. We used the same experimental setup again in 2018, to study pathogen progression in the queen (quantification by qPCR), in the solitary colony founding phase (yellow), as well as the first worker phase (green), hibernation (blue) and 1 year after flight (purple). At this time, we also assessed long‐term transgenerational immunisation effects of the queen towards her worker offspring by determining worker disease susceptibility to the same pathogen. Sampling points are depicted as vertical bars

Queens of the black garden ant, *L*.* niger*, were collected after nuptial flights in July 2014 and 2018 in Klosterneuburg, Austria. As *M*. *brunneum* is a natural pathogen in our study population (Pull & Cremer, 2017), we monitored *Metarhizium*‐induced mortality in field‐collected queens in 2018 and found that 1.5% of the queens died from natural *Metarhizium* infections within 1 week after flight (detailed in SI). While high *Metarhizium* doses induce disease, lower doses can lead to asymptomatic infections (Konrad et al., 2012), which can persist in *L*. *niger* queens for longer periods (Pull et al., 2013), so that a proportion of the queens surviving the first week was expected to carry natural low‐level field infections of *Metarhizium*. One week after mating (Table S1), we exposed individual queens to either a sham treatment of 0.05% sterile Triton‐X or a very low dose of *M*. *brunneum* (application of a 0.5 μL droplet of 2×10^4^ conidiospores/mL in 0.05% sterile Triton‐X with confirmed germination rate >95%, equivalent to 10 conidiospores/queen; strain Ma275 obtained from the University of Copenhagen, KVL 04‐57). This low‐dose pathogen treatment was expected to induce acute queen mortality in comparably low levels as natural infection, and sublethal infections in several of the exposed queens, whilst sham‐treated queens should only experience natural infection levels. Queens and their developing colonies were reared at 23°C for 1 year, except for a 20‐week winter period at 4°C and observed for colony development in 2014 and in 2018 for queen pathogen load proliferation and worker immunity 1 year after queen flight (Figure 1 and SI). Although founder mass is a strong predictor of resource availability in termites (Cole et al., 2018; Cole & Rosengaus, 2019), initial queen mass following the mating flight does not predict brood production nor antifungal activity of the queens in our study population (CDP, personal communication), and was not measured in this study to avoid causing unnecessary stress, which may have long‐lasting effects itself (Bordoni et al., 2017).

### Colony developmental trajectory

In 2014, we recorded colony size and caste composition by monitoring the numbers of workers, pupae and large (>3rd instar) larvae, as well as presence/absence of smaller larvae and eggs in colonies established by pathogen‐ and sham‐treated queens (*n* = 24 each; for sample sizes per timepoint see Figure S1). We filmed (cameras IDS UI‐1640LE, software StreamPix, at 15–18 fps) each colony in 3 h sessions, at four time points before, and five time points after hibernation (when containing at least five workers; no recording during hibernation). We monitored queen survival and determined colonies had failed hibernation when the queen died during winter, or their colonies collapsed to less than five workers followed by queen death within the next 8 weeks. We acquired behavioural data from 312 videos (190 before and 122 after hibernation, as 25 sampling points could not be analysed for biological or technical issues; Figure S2). Videos were analysed with customised tracking software (Naiser et al., 2018), using the IST Austria High‐Performance Computing cluster to obtain ant motion and positional data, to derive three colony behaviour metrics: (1) locomotion activity (proportion of workers moving), (2) foraging activity (proportion of workers foraging) and (3) worker aggregation around the queen (proportion of workers near the queen; Figure S2a). Metrics were condensed to a single mean value per filming session.

### Queen pathogen progression and worker pathogen susceptibility

In 2018, we similarly set up 270 pathogen‐treated queens to monitor pathogen progression over the course of colony founding, and 72 sham‐treated queens as controls. At 11 timepoints––spanning the solitary queen phase, first worker emergence, hibernation and mature colony phase (Figure 1, Table S1)––we randomly sampled a subset of colonies per treatment (Table S2), counted workers and brood and quantified the queen's pathogen load by quantitative real‐time PCR (qPCR) targeting the *Metarhizium*‐specific sequence of the fungal ITS2 region (Giehr et al., 2017; Stroeymeyt et al., 2018). After 1 year, we determined worker susceptibility to the pathogen their mother was exposed to a year before, by challenging individual workers from 1‐year‐old colonies (15 established by pathogen‐ and 15 by sham‐treated queens), to either a sham (Triton X, *n* = 126), a high (~30,000 conidiospores/worker, *n* = 123) or a very high (~150,000 conidiospores/worker, *n* = 122) load of the same strain of *M*. *brunneum* (by applying 0.3 μl of a 1 × 10^8^ resp. 5 × 10^8^ conidiospores/mL suspension). We monitored worker survival daily for 12 days. Dead workers were surface sterilised and observed for *Metarhizium* outgrowth.

### Statistical data analysis

All analyses including testing for model assumptions and stability (‘DHARMa’, Hartig, 2021) were performed in R‐v.3.6.3 (R Core Team, 2020), as detailed in the SI. We mostly tested our model predictors and their interactions using generalised linear mixed models (GLMMs; ‘lme4’ Bates et al., 2015; ‘glmmTMB’ Brooks et al., 2017) by comparison of null models with full or reduced models (Bolker et al., 2009). When interactions were non‐significant, we removed them from the models, when significant, we continued by direct comparison of the treatment groups by separate models or direct post hoc testing (‘emmeans’ Lenth, 2019; ‘multcomp’ Hothorn et al., 2008). Logistic regressions were implemented as GLMs with binomial error distribution and logit link function. In case of multiple testing, we applied the Benjamini–Hochberg procedure to protect against a false discovery rate of 5% (Benjamini & Hochberg, 1995) and report two‐sided, corrected *p* values. We used ‘ggplot2’ (Wickham et al., 2018) for graphs.

We examined how colony worker number was affected by queen treatment (pathogen/sham), experimental week and pre‐/post‐winter period, and their interactions by a GLMM, including random intercepts, random slopes and their interaction. We compared regression slopes (i.e. colony growth) between groups (pathogen/sham) fitting a regression for pre‐/post‐winter period. For the successfully hibernating colonies we compared growth rates before/after winter with a linear model, and used unpaired Wilcoxon signed‐rank tests to compare worker numbers between groups at different time points. We tested with logistic regressions whether hibernation success depended on queen treatment, and the composition and behavioural state of the colony before hibernation (week 18). We performed repeated‐measures correlations between behaviours and pupal as well as worker number. We performed logistic regression to test if presence/absence of detectable queen pathogen load was predicted by reproductive investment (pupal number) and time (week), or their interaction, and evaluated how pathogen proliferation (pathogen load) depended on pupal number by linear regression. Cox proportional‐hazards (‘survival’ Therneau, 2021; ‘survminer’ Kassambara et al.., 2021), were used to (i) analyse queen survival depending on their treatment (pathogen/sham) and (ii) to test whether survival of workers from 1‐year‐old colonies depended on the combination of their maternal treatment (pathogen/sham) and worker challenge (sham, high and very high), after having checked for the absence of a difference in colony size and worker winter mortality (see SI). We included colony as a cluster term to account for non‐independence of workers from the same colony (Therneau & Grambsch, 2000), and compared the two worker sham treatments by log‐rank test due to the absence of mortality in one of these groups. Worker mortality over winter was analysed by logistic regression. The SI contains further analyses on the effect of queen treatment on colony brood production and behaviour.

## RESULTS

### Queen pathogen exposure affects colony growth

**FIGURE 2:**
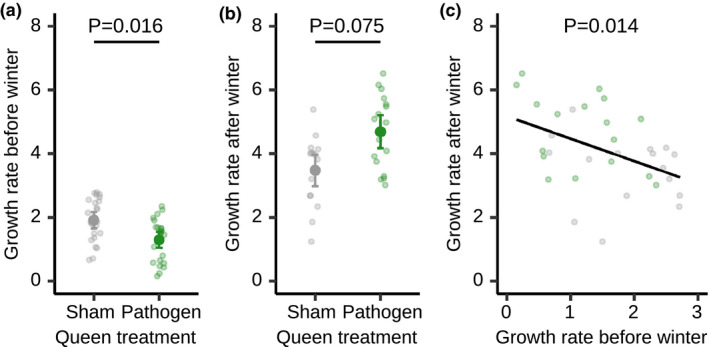
Pathogen exposure reduces growth rate before winter but colonies recover in size after hibernation. Growth rates depended on the interaction between queen treatment and pre‐ versus post‐hibernation period; (a) pathogen treatment reduced growth rate before winter, whereas (b), after winter the surviving pathogen‐treated (green) colonies had more than tripled their growth rate and were marginally growing faster than the surviving sham‐treated colonies (grey). (c) For both treatments, surviving colonies that grew slowly before winter, grew faster afterwards and vice versa. Individual data points shown along with (a, b) means +/‐ 95% CI, (c) regression line, *R* ^2^ = 0.19. Based on *n* = 48 colonies, 24 each developing from a pathogen‐ or a sham‐treated queen, collected in 2014. See Table S3 and Figure S1 for further detail

Queen low‐level exposure to the fungal pathogen *M*. *brunneum* affected the colony growth trajectories. Worker number depended on queen treatment in relation to the week of the experiment and whether the observation was pre‐ or post‐hibernation (Figure 2a,b [LMER] overall model LR *Χ* ^2^ = 326.64, df = 7, *R* ^2^cond = 0.867, *p* < 0.001; interaction LR *Χ* ^2^ = 22.554, df = 2, *p* < 0.001). Colonies founded by pathogen‐treated queens grew at a slower rate before winter compared to control colonies (Figure 2a [LMER] overall model LR *Χ* ^2^ = 80.28, df = 3, *R* ^2^cond = 0.916, *p* < 0.001; queen‐treatment × time interaction LR *Χ* ^2^ = 7.04, df = 1, *p* = 0.016). After winter, pathogen‐treated colonies showed a non‐significant trend of a faster growth rate (Figure 2b [LMER] overall model LR *Χ* ^2^ = 54.92, df = 3, *R* ^2^cond =0.907, *p* < 0.001; queen‐treatment × time interaction LR *Χ* ^2^ = 3.15, df =1, *p* = 0.075), indicating that they could compensate for their slower early growth after hibernation, so that colony sizes after 1 year were indistinguishable between treatments (Figure S1 [Wilcoxon signed‐rank test] V = 85.5, *p* = 0.407). Interestingly, for all colonies surviving hibernation, independent of queen treatment, colony growth before winter negatively predicted their after winter growth rate (Figure 2c [LM] F = 27.89, df = 1,31, *R* ^2^ = 0.194, *p* = 0.014), revealing a general trade‐off between pre‐ and post‐winter growth, where fast starters grew more slowly after winter, while slow starters increased their rate after winter.

### Hibernation success depends on low pre‐winter brood investment

**FIGURE 3:**
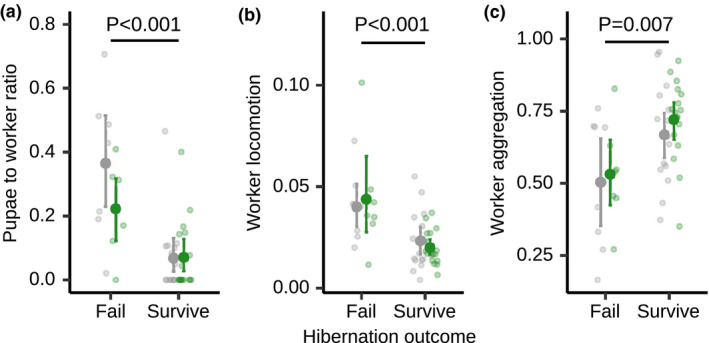
Successfully hibernating colonies show low pre‐winter brood investment, low worker locomotion and high aggregation around the queen. (a) Colonies that survived overwintering had a lower number of pupae with respect to the number of workers before hibernation (week 18), compared to colonies which failed, regardless of whether they were founded by pathogen‐treated (green) or control queen (grey). Surviving colonies also had (b) lower worker locomotion and (c) higher worker aggregation around the queen. All data points, mean and 95% CI are displayed. Based on *n* = 48 colonies, 24 each developing from a pathogen‐ or a sham‐treated queen, collected in 2014. See also Figures S2, S3

The harsh winter conditions in our experiment caused high colony mortality during hibernation, with 35% of the queens (9/24 pathogen‐ and 8/24 sham‐treated queens) dying. Hibernation success was neither predicted by queen treatment nor overall colony pre‐winter growth rate, but was highly dependent on colony composition, namely the pupae‐to‐worker ratio shortly before winter (week 18; Figure 3a [GLM] overall model LR *Χ* ^2^ = 18.8, df = 3, Tjur's*R* ^2^ = 0.383, *p* < 0.001; queen treatment LR *Χ* ^2^ = 0.03, df = 1, *p* = 0.851; colony growth rate LR *Χ* ^2^ = 0.01, df = 1, *p* = 0.893; pupae‐to‐worker‐ratio LR *Χ* ^2^ = 17.43, df = 1, *p* < 0.001; model derivation detailed in SI). This means that queen survival was improved when the number of pupae each worker had to help rear was lower. Before winter, colonies had reached an average of seven workers per pupae (pupae‐to‐worker ratio 0.14, sd = 0.18). Such an average colony was approximately 80% more likely to survive hibernation than colonies that only had three workers available to rear each pupa (pupae‐to‐worker ratio = 0.32, hazard ratio HR = −1.67).

### Successfully hibernating colonies are less active and more aggregated before winter

We monitored colony behaviour, in particular, the proportion of workers foraging and showing locomotor activity, or aggregating around the queen over colony development, using automatised tracking software on our video data (Figure S2a). Foraging only occurred in relevant numbers after winter and all colony behaviour was highly dynamic over the year, but was independent of queen treatment (all *p* > 0.3, Table S5, Figure S2b‐d). The proportion of workers foraging or showing active locomotion was independent of colony size (repeated‐measures correlations; both *p* > 0.2), whilst the proportion of workers aggregating around the queen was slightly decreasing with increasing colony size (r = −0.157, df = 271, *p* = 0.027; Figure S3). Interestingly, hibernation success could be predicted by worker activity; successfully hibernating colonies were characterised by a lower worker locomotion (Figure 3b [GLM] overall model LR *Χ* ^2^ = 15.47, df = 2, Tjur's*R* ^2^ = 0.303, *p* < 0.001; locomotion: LR *Χ* ^2^ = 15.38, df = 1, *p* < 0.001) and higher worker aggregation around the queen before winter, compared to those later failing during hibernation (Figure 3c [GLM] overall model LR *Χ* ^2^ = 10.69, df = 2, Tjur's*R* ^2^ = 0.207, *p* = 0.008; aggregation: LR *Χ* ^2^ = 9.97, df = 1, *p* = 0.002). Moreover, the number of pupae present in the colonies was strongly correlated with both worker locomotion and aggregation. The more pupae a colony had, the higher the locomotor activity of its workers (repeated‐measures correlation: *r* = 0.54, df = 271, *p* < 0.001) and the lower their degree of aggregation near the queen (*r* = −0.55, df = 271, *p* < 0.001; Figure S4). Automated monitoring of colony behaviour therefore revealed that colony composition, especially a colony's brood care needs, was mirrored in worker behaviour, which strongly affected hibernation outcome.

To investigate why a large number of pupae paired with a small worker force so greatly affected queen hibernation survival, we extended our study to assess the queen's ability to suppress fungal infection over the course of the resource‐demanding phase of colony founding, starting with the queen solitary phase before worker emergence, during which all brood care is performed by the queen alone.

### Queens producing many pupae suffer higher pathogen loads in the solitary phase

**FIGURE 4:**
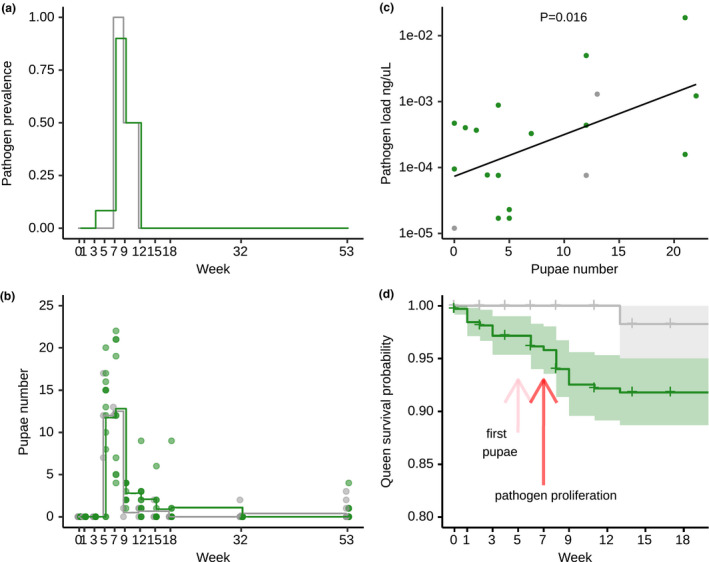
Queen pathogen load and mortality peaks after production of queen‐reared pupae. (a) The proportion of live‐sampled queens with detectable pathogen load was very low at most sampling times over the course of their first year of colony founding, but peaked in weeks 7–9 for both pathogen‐ (green) and also sham‐treated (grey) queens, where we observed three natural infections (two out of two sampled in week 7 and one out of two sampled in week 9). Queen pathogen load was hence increased in the period following the peak of production of pupae (b) in the queens’ solitary phase (mean shown as step line, darker dots correspond to multiple overlapping data points). (c) Queen pathogen load (measured in ng/µl by qPCR quantification) was higher, the higher the number of pupae produced until the sampling time point (linear regression line and individual data points of the 16 pathogen‐ and 3 sham‐treated queens with detectable load displayed). (d) Survival of pathogen‐treated queens decreased slightly in the acute phase shortly after low‐level exposure with *M*. *brunneum*, and showed a strong decrease at the end of their solitary phase, following the peak of pupal production (see b), whilst sham‐treated queens had a high survival before winter (95% CI area indicated by shading around the survival curves, tick marks indicate censored data, i.e. queens sampled for pathogen quantification); based on the queens from the 2018 collection, with a total of *n* = 183 pathogen‐ and *n* = 65 sham‐treated queens sampled alive, and *n* = 24 and *n* = 32 pathogen‐treated queens dying before and during winter, respectively, as well as *n* = 1 and *n* = 3 sham‐treated queens dying before and after winter (for detailed sampling regime see Table S2)

We monitored pathogen progression in the queens during their solitary colony founding phase, after first worker emergence, during hibernation and 1 year after mating (Figure 1) by qPCR of the *Metarhizium*‐ITS2 gene (as detailed in SI). Since we exposed the queens to a very low dose that is below the detection threshold of the qPCR method, a positive PCR result indicated active fungal proliferation inside the host. Fungal prevalence in live‐sampled queens peaked in weeks 7 and 9 after queen flight for both queen treatments (Figure 4a), directly following the production of first pupae in weeks 5 and 7 (Figure 4b). This suggests that the queen's past reproductive investment in the first brood cycle may negatively impact her ability to fight pathogen replication. Our sensitive qPCR method also detected *Metarhizium* in three control queens, at––and only at––the period following high brood investment. This suggests that also naturally existing low‐level infections with this pathogen, which is a common sympatric pathogen in our host study population (see methods) known to induce persistent sublethal infections in *L*. *niger* queens (Pull et al., 2013), could no longer be suppressed by the queen immune system.

For individual pathogen‐treated queens, we found that the more pupae a queen had produced at the time of sampling, the higher the probability that the pathogen had replicated in her body ([GLM] LR *Χ* ^2^ = 11.15, df = 1, *p* = 0.002). For all pathogen‐treated queens with detectable pathogen load, pupal number positively predicted the level of fungal replication in her body ([LM] LR *Χ* ^2^ = 5.1, df = 1, *R* ^2^ = 0.268, *p* = 0.039), a relationship that was equally found when including the three naturally infected queens with detectable pathogen load (Figure 4c [LM] LR *Χ* ^2^ = 7.26, df = 1, *R* ^2^ = 0.333, *p* = 0.016). This suggests that, in general, by the end of the solitary period, experimentally induced or naturally acquired infections could no longer be effectively suppressed by the queen's immune system, and that this effect was stronger, the larger the queen's reproductive investment. Interestingly, once workers had emerged, no queen had detectable *Metarhizium* levels, indicating that the queens were again able to suppress pathogen proliferation when workers had taken over brood care, that is, when colony founding had switched from the individual queen phase to the superorganism phase. Only one pathogen‐treated queen that died during winter showed a detectable infection, suggesting queens were not necessarily able to clear their low‐level infections before winter. Lastly, after 1 year, when the colonies had reached their ergonomic phase, no *Metarhizium* was detected in any of the queens.

### Pathogen‐induced queen mortality occurs both in the acute and chronic infection phases

Pathogen treatment led to a higher mortality in queens compared to the sham treatment (Figure 4d; Cox proportional‐hazards regression, LR *Χ* ^2^ = 4.68, df = 1, *p* = 0.030); in the acute phase of infection within the first month after exposure, the pathogen had a killing rate of 2.8% (CI 1.0%–4.7%) for the pathogen‐treated queens, with no deaths in the controls (Figure 4d). Our long‐term survival analysis revealed a second wave of pathogen‐induced killing, with a mortality risk of 8.2% (CI 5.0%–11.3%) for the pathogen‐treated queens and 1.7% (CI 0.0%–5.0%) in the controls. This second‐period mortality is concurrent with the highest fungal prevalence in the live‐sampled queens (weeks 7–9), towards the end of the queen solitary phase (Figure 4a,d); in total 10/24 pathogen‐treated queens dying before winter showed detectable pathogen load immediately after death, whilst the one dying sham‐treated did not. Queen mortality over hibernation was similar to the first experiment, with an overall 26% of the queens dying, and independent of queen treatment (32/107 of the pathogen‐treated and 3/26 of the sham‐treated queens died, out of which only a single, pathogen‐treated queen had detectable pathogen load; [GLM] LR *Χ* ^2^ = 2.66, df = 1, *p* = 0.103). Worker hibernation survival was similarly unaffected by the treatment of their queen ([GLMER] LR *Χ* ^2^ = 0.142, df = 1, *p* = 0.706). Among the colonies in which the queen successfully overwintered, approximately 70% of the workers did not survive winter, with a mean of 12.7 (CI 10.8–14.6) workers dying in pathogen‐treated colonies versus 11.6 (CI 3.2–20) in the sham‐treated ones. After hibernation, no additional queens died.

### Workers are less susceptible to a pathogen that their mother experienced 1 year earlier

**FIGURE 5:**
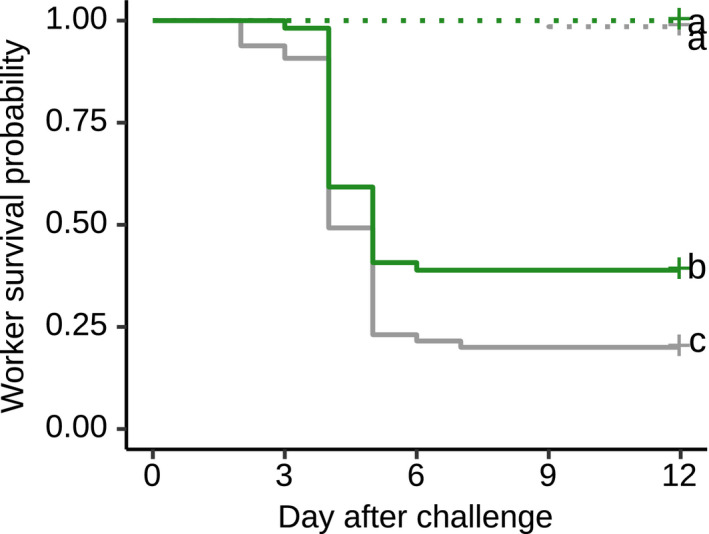
Transgenerational immunisation of workers 1 year after queen pathogen exposure. Workers from 1‐year‐old colonies were better able to survive a challenge with a high dose (30,000 conidiospores/worker) of the same *M*. *brunneum* strain encountered a year before by their mothers in a low dose (green line) compared to workers from sham‐treated queens (grey line). This indicates a protective transgenerational effect of the pathogen experience of the mother on the disease susceptibility of her worker offspring. There was no difference in survival of offspring of pathogen‐treated (green dotted line) and sham‐treated (grey dotted line) queens in the absence of a pathogen challenge of the workers (worker sham treatment; log‐rank test *Χ* ^2^ = 24, df = 27, *p* = 0.6). Different letters denote significant post hoc comparisons (*p* < 0.05). Based on workers from 13 pathogen‐ and 15 sham‐treated queens, collected in 2018 (worker sham treatment: *n* = 56 workers from pathogen‐ and *n* = 68 from sham‐treated queens, out of which one died without any later *Metarhizium* outgrowth; worker high pathogen exposure: *n* = 54 from pathogen‐ and *n* = 65 from sham‐treated queens, out of which 85 died and 82 [96%] also showed *Metarhizium* outgrowth). See also Figures S1, S5

We tested whether the queen's early‐in‐life pathogen experience influenced the disease susceptibility of her developing workforce, by challenging workers 1 year after queen treatment with either a high (~30,000 conidiospores) or a very high (~150,000 conidiospores) dose of the same pathogen, compared to a sham control worker treatment. Worker mortality was virtually absent after sham treatment, but increased drastically after pathogen exposure. Workers receiving a high pathogen challenge were 38% less likely to die when their mother had been exposed to the same pathogen a year before compared to offspring of sham‐treated queens (Figure 5; Cox proportional‐hazards regression, overall model Wald = 38.5, df = 4, *p* < 0.001; post hoc comparison between workers from pathogen‐ vs. sham‐treated queens after high exposure *p* = 0.014; hazard ratio HR = 0.62, CI 0.43–0.88). When workers were exposed to a very high pathogen challenge, however, maternal pathogen experience did no longer provide a protective effect and mortality was equally high for offspring of pathogen‐ and sham‐treated mothers (Figure S5; *p* = 0.82). Together this revealed that maternal pathogen experience had no influence on worker offspring survival in the absence of or under an overwhelmingly high pathogen challenge, yet that at less overwhelming challenge levels maternal low‐level exposure to the same pathogen a year before made her worker offspring less susceptible to disease.

## DISCUSSION

To determine the long‐term impact of early disease stressors on superorganism developmental trajectories and immunity in the social Hymenoptera, we followed *L*. *niger* ant queens through their first year of colony founding. In contrast to *L*. *niger* queens that were experimentally exposed to soil borne pathogens (Tragust et al., 2020), but similar to *Metarhizium* exposure of termite founding pairs (Calleri II et al., 2006; Cole et al., 2018) or co‐founding *L*. *niger* queens (Pull et al., 2013), we found a fecundity‐immunity trade‐off (Schwenke et al., 2017)⁠, as low‐level *M*. *brunneum* queen exposure slowed down early colony growth. Following the colonies over a whole year, however, showed that these early effects on colony growth were transient, again akin to termite colony development (Calleri II et al., 2006), as pathogen‐treated colonies reached similar sizes than control colonies after winter. Similar trajectories have also been found in unitary organisms (Metcalfe & Monaghan, 2001). Additionally, we found that, independent of queen treatment, colonies growing fast before winter slowed down after hibernation, and *vice versa*. Fast early growth was, therefore, not the only trajectory to achieve large mature colony sizes, which ultimately increases competitiveness against other colonies (Dornhaus et al., 2012; Sommer & Hölldobler, 1995) and assures high reproductive output (Shik, 2020).

Colony size before winter did not affect the chances of a colony failing during hibernation. Instead, the pace at which the colony produces its workforce strongly predicted overwintering success: only when a ratio of three workers to every pupa was surpassed did the chances of overwintering survival improve. While this relationship may have been affected by the harsh winter conditions in our experiment, mature colonies also show highest rearing success with a worker‐to‐pupae ratio of at least 1:1 (Purcell et al., 2012). Interestingly, hibernation success was equally well predicted by taking a snapshot of a colony's behaviour before winter. Automatised video analysis revealed that colonies failing during winter had higher pre‐winter worker locomotion and lower worker aggregation around the queen. As workers did not forage before winter, this behavioural pattern likely reflects brood care‐related activity. Higher aggregation near the queens that later survived winter could also indicate more worker–queen interactions, such as feeding and grooming, and may also benefit thermoregulation (Perez & Aron, 2020), which could all improve queen condition and thus overwintering survival.

Our work reasserts that low pathogen exposure early‐in‐life can lead to persistent infections in ant queens that last multiple weeks. While initial exposure levels were below our PCR detection limit, we observed subsequent pathogen replication in the queens over the course of colony foundation. Monitoring queen pathogen load showed that replication was most prominent towards the end of the queen's solitary colony founding phase, in which she rears all brood alone by metabolising fat and certain tissue reserves. Queens that produced the most pupae in this phase bore the highest pathogen loads, presumably as they invested more heavily into brood production and leaving fewer resources available for immunity (Pull et al., 2013). Many queens harboured persistent fungal infections (established either from experimental exposure or natural field infections) that were not cleared, but suppressed to asymptomatic levels, when the queens had plenty of resources at the beginning of the solitary phase (Keller & Passera, 1989) or when workers began monopolising brood care. Towards the end of the solitary phase, however, the queen's immune system likely becomes compromised due to investment into brood care, no longer being able to suppress the pathogen. This supports the notion that a fecundity–immunity trade‐off is most critical when queens have depleted their bodily reserves and have yet to establish a workforce (Tragust et al., 2020).

While low‐level infections with *Metarhizium* in *Lasius* are typically asymptomatic (Konrad et al., 2012), immunosuppression may permit persistent infections to develop into disease (Kennedy, 2020), which leads to host death with obligately killing *Metarhizium* (Thomas & Read, 2007). Indeed, during the late solitary phase, pathogen‐treated queens had a much higher risk of dying than sham‐treated queens, which was even more pronounced than during the acute infection phase directly after exposure. Winter mortality was independent of queen treatment in our experiment, whereas Tragust et al., (2020) found that pathogen‐exposed *L*. *niger* queens suffered higher later mortality than unexposed queens, when their workforce was experimentally removed. Together, this suggests that queens harbouring persistent infections may rely more strongly on their workers to survive stressful periods compared to healthy queens. A proximate explanation may lie in the interplay between hormonal regulation of larval maturation (Hiruma & Kaneko, 2013) and diapause (Denlinger et al., 2012) with the immune system in insects, where key regulatory compounds, such as juvenile hormone (JH), are known to suppress immunocompetence (Schwenke et al., 2017). In ants, JH is transferred to larvae via workers (LeBoeuf et al., 2016) and potentially by the queen, who rears the first larvae and continues to influence larval development once workers emerge (Kipyatkov et al., 1996). We hypothesise that it may be particularly risky for queens carrying chronic infections to produce high levels of JH to regulate brood development and winter‐induced diapause, and these queens would thus profit most from ‘outsourcing’ this task to workers.

While early‐in‐life pathogen exposure of the queen did not strongly impact long‐term colony development, we found that it equipped workers with an increased ability to survive exposure to the same pathogen that their mother experienced 1 year before, except under an overwhelmingly high pathogen dosage. Our dosage experiment thus suggests that workers will be protected when encountering infectious stages of *Metarhizium* in contaminated environments, such as soil known to contain up to 5,000 conidiospores per gram (Cremer et al., 2018), but contact with highly infectious and spore‐laden sporulating cadavers may still cause disease. Previous pathogen exposure can reduce future susceptibility to the same pathogen, both within the lifetime of an individual or as a transferred effect in offspring, respectively, termed immune priming and transgenerational immune priming in invertebrates (Contreras‐Garduño et al., 2016; Kurtz & Armitage, 2006; Kurtz & Milutinović, 2016; Masri & Cremer, 2014). Immunisation of individuals following pathogen contact has been demonstrated in ants before, both across castes and developmental stages (larvae: Rosengaus et al., 2013, workers: Konrad et al., 2012; queens: Galvez & Chapuisat, 2014) and transgenerational immunological effects were also found: queen pathogen exposure was shown to protect her larvae (Fuchs et al., 2018) and first workers (Bordoni et al., 2018), mirroring findings in bees and termites (Cole et al., 2020; Sadd et al., 2005; Salmela et al., 2015). However, here we found the protective effect of maternal transgenerational immunisation to be remarkably long lasting, protecting the queen's workforce for at least 1 year. Transgenerational immune priming is known to be costly and so causes trade‐offs such as increased susceptibility of the offspring to other diseases (Sadd & Schmid‐Hempel, 2009). In our study, we could not detect any survival costs due to transgenerational protection, as mortality of sham‐treated workers from 1‐year‐old colonies was unaffected by maternal exposure, and there was no difference in hibernation mortality of workers from pathogen‐ versus sham‐treated mothers. Ant colonies are general sessile (Hakala et al., 2019) and so expected to experience a stable pathogen community over time, such that repeated pathogen encounters may favour the transferal of lasting immunity to offspring (Roth et al., 2018; Tetreau et al., 2019). Such enduring immunological effects may be particularly rare though in other insects due to their shorter lifespans, making long‐lived species like ants (Kramer et al., 2016) a valuable model system to study the mechanistic basis of long‐lasting immunisation.

Currently, the mechanisms underlying the long‐term protection we observed are still elusive. Maternally transmitted transgenerational immune priming can occur through vertical transfer of microbial antigens into eggs, inducing an immune response in the offspring, or by epigenetic effects via DNA methylation, histone modification or microRNAs (Vilcinskas, 2021). As recently shown for the honeybee, workers can contribute to microbial antigen transfer during offspring rearing through the royal jelly (Harwood et al., 2021), providing horizontal protection. Given the generational overlap between the queens and her offspring and that the liquid food they exchange contains a multitude of compounds including microRNAs (LeBoeuf et al., 2016), we suggest that it may be possible for the queen to horizontally transfer immune eliciting or modulating compounds during larval feeding. This may be further facilitated by the fact that many queens did not clear but had established chronic low‐level infections over long periods. It is also unclear if the reduced susceptibility of the workforce in 1‐year‐old colonies was achieved through the queen protecting just the first workers that emerged––implying that these workers maintained immunisation over several months––or whether the queen continued to confer protection on newly emerged workers, many months after her own pathogen exposure. Our data suggest the latter, as approximately 70% of the first workers of each queen died during hibernation. As colonies only become sexually reproductive (producing dispersing daughter queens and sons) one to several years after colony founding, such a long‐lasting ability of the queen to transfer immunity could also provide a mechanism to immunise not only her own colony by immunising her worker offspring, but even the next generation of colonies by immunising the dispersing sexuals, leading to transgenerational protection of new superorganisms.

## CONCLUSION

By following the long‐term development of ant colonies, we show that superorganismal development, similar to organismal development, is characterised by trade‐offs between immunity and reproduction. On the one hand, workers help to relieve the queen of some reproductive tasks and hence partly free her from the constraints of a fecundity‐immunity trade‐off (Calleri II et al., 2006; Cole et al., 2018; Schwenke et al., 2017; Tragust et al., 2020). On the other hand, the queen can transfer her own pathogen experience to offspring via transgenerational immunisation, which generates highly durable disease protection in the workers. Importantly, as the worker offspring do not disperse but remain with their mother, this transgenerational protection positively feeds back into protection of the colony, and the queen herself. In other words, long‐term maternal transgenerational effects on worker disease susceptibility leads to the immunological protection of the soma after pathogen exposure of the germline, and hence long‐lasting immunity of the whole superorganism.

## CONFLICT OF INTEREST

The authors declare no competing interests.

## AUTHORSHIP

BCP, CDP and SC designed the study. BCP, CDP and EN performed the experiments. FN, BCP and JM adapted the tracking software for the specifics of this project. BCP analysed the data. BCP and SC wrote the manuscript with input from CDP. All authors critically revised and approved the manuscript for publication.

## ETHICS

*Lasius niger* is not a species listed under regulation or protection status according to the Office of the Federal Government of Lower Austria, in particular, the Department for Nature Protection; its collection and all experimental work performed followed the European and Austrian laws and institutional ethical guidelines, including observation of the Nagoya Protocol on Access and Benefit Sharing.

### PEER REVIEW

The peer review history for this article is available at https://publons.com/publon/10.1111/ele.13907.

## Supporting information

Supplementary Material

## Data Availability

All data files are available at Dryad Digital Repository under https://doi.org/10.5061/dryad.7pvmcvdtj. Documentation and source code to compute the behavioural metrics from motion trajectories are provided on GitHub (https://github.com/wildflowersbloom/colony‐development), as well as the motion tracking software (https://github.com/Flipajs/FERDA), both code repositories under an MIT open‐source licence.
