# Supplementary material for: Early queen infection shapes developmental dynamics and induces long‐term disease protection in incipient ant colonies
Source: Ecol Lett. 2021 Nov 1;25(1):89–100. doi: 10.1111/ele.13907 (PMC9298059; doi:10.1111/ele.13907)
Supplement: Supplementary file 1 — Supplementary Material [file ELE-25-89-s001.pdf]

SUPPLEMENTAL INFORMATION

# Early queen infection shapes developmental dynamics and induces long-term disease protection in ant colonies

Barbara Casillas-Pérez, Christopher D. Pull, Filip Naiser, Elisabeth Naderlinger, Jiri Matas, Sylvia Cremer

## SI METHODS

### Experimental methods

*Ant queens and fungal pathogen.* We collected several hundred newly mated queens of the black garden ant, *Lasius niger,* in both July 2014 (for assessment of colony long-term development after early queen pathogen exposure) and July 2018 (for queen pathogen progression and the immunity of their workforce) after their nuptial flights and before they burrowed into the soil in Klosterneuburg, Austria (48.3095,16.2598 DD). *L. niger* is a very common species in the study population and mating flights consist of thousands of sexuals, all triggered by species-specific microclimatic conditions, synchronising dispersal from their natal colonies (Hart *et al.* 2018), assuring mixing between colonies and inbreeding avoidance (Hakala *et al.* 2019). Colony identity was thus not traceable for collected queens, but given their massive numbers in the mating flights, the probability of two queens stemming from the same maternal colony is considered minimal, corroborated also by the fact that if two queens of this species co-found a nest after the mating flight, they are typically unrelated (Bernasconi & Strassmann 1999). We determined natural infection load of the queens in our study population in 2018, and found that tachinid flies emerged from 1.3% (7/526) of the queens and that 2.3% (12/526) of the queens died within one week after flight followed by fungal outgrowth, two thirds of which (8/12) showed positive results for a quantitative real-time PCR with *Metarhizium*-specific primers (Giehr *et al.* 2017, detailed below).

This revealed (i) that *Metarhizium* is a sympatric pathogen in our study population and (ii) that it causes natural infections inducing mortality directly after flight in 1.5% of the queens. As it is known that low-level infections with *Metarhizium* can remain asymptomatic (Konrad *et al.* 2012), and infections with *Metarhizium* can persist in *L. niger* queens for a number of weeks (Pull et al. 2013), several of the queens surviving the first week and establishing a colony were expected to be infected with sub-lethal natural infections of *Metarhizium* in our experiments.

To simulate the effect of queens becoming exposed to this sympatric, natural fungal pathogen while digging into contaminated soil during colony founding, we experimentally exposed the freshly collected queens to a low dose of *M. brunneum* infective stages (conidiospores) or a sham control, and compared colony development, queen pathogen progression over the course of her solitary and social founding phase, as well as long-term immunity of the colony for one year. Specifically, we exposed queens within a week after flight to *M. brunneum* (strain Ma275 KVL 04-57, obtained from the University of Copenhagen), that had been cultivated on sabaroud dextrose agar plates at 23°C. Conidiospores were harvested in 0.05% sterile Triton X and were confirmed to have a germination rate >95%. Ma275 causes high mortality in *L. niger* queens when applied in a high dose (Pull & Cremer 2017), yet we exposed the queens to a low dose of 0.5 µL of $2\times10^4$ conidiospores/mL in 0.05% sterile Triton-X (equivalent to 10 infective conidiospores/queen), by holding each queen with sterile soft forceps and pipetting a droplet of the treatment suspension onto her thorax. Similar to low-dose treatments of *L. neglectus* ants with the same fungal strain regularly establishing low-level infections at doses inducing very low mortality (approx. 2%; Konrad *et al.* 2012), we here tried to obtain low acute queen mortality in comparable levels to natural *Metarhizium*-induced queen death after flight (approx. 2%), which we expected would equally induce sub-lethal infections in several of the exposed queens. Control

queens were subjected to a sham treatment of 0.05% sterile Triton-X only. After treatment, the queens were reared in individual vials with a moist plaster of Paris substrate (height=3cm, Ø=2cm) without food until the first workers emerged. Then, colonies were transferred to petri dishes with two compartments (height=1.4 cm, Ø=9 cm): one half was plaster-covered and regularly moistened and used by the ants as the nest area, whilst the other, unplastered half was used as a foraging arena, in which we placed a vial of sucrose solution (25%) and minced cockroach (*Blaptica dubia*). This setup allowed us to clear food and debris with minor disturbance to the colony. Colonies were kept in a climate-controlled room at 23°C and 70% humidity and a 12:12 h light:dark schedule for the experimental periods before and after hibernation. For hibernation, the colonies were placed into an incubator with constant 4°C in week 19 of the experiment, in which they remained for a 20-week period from mid-November to mid-April, before being placed back into the climate-controlled room. We applied this procedure to 24 pathogen- and 24 sham-treated queens in 2014 to obtain longitudinal data on colony development in 2014 and repeated it in 2018 with 270 pathogen- and 72 sham-treated queens to get data on pathogen load proliferation in the queens, and test worker immunity one year after queen flight (Table S1).

*Filming.* With the colonies established from the 2014 collection, we filmed three-hour-long sessions of the colonies at nine time points throughout the year, starting when at least five workers had emerged (10 weeks after mating flight; average 10.4±3.8 workers), as detailed in Figure 1 and Table S1. We analysed the videos by motion tracking to infer worker activity (locomotion and foraging) and worker aggregation near the queen. Half an hour before filming, we removed food, water and large debris from the dish, and added a droplet of honey, sandwiched between paper towel and plastic, to use as a clearly delineated foraging area (approx. 2 $cm^2$). We recorded four

colonies at a time, from two sham- and two pathogen-treated queens, in a parallel setup (rolling-shutter cameras from IDS UI-1640LE USB 2.0 CMOS, 1024x1024 Pixel, 1.3” Aptina Sensor, rolling-shutter; fixed focal length lense 6MM 1/1.8” f 1.4-f/16 C-mount, Edmund Optics; Streampix software for acquisition) at 15fps before winter and 18fps after winter conditions. This frame rate increase was motivated by achieving better trajectories, yet pre-winter videos were not substantially more fragmented. All three-hour sessions were filmed between 7am and 8pm and the order in which colonies where filmed was randomized at each timepoint.

We analysed 190 videos out of 192 filmed before hibernation (i.e. 48 colonies alive at 4 timepoints), as 2 were unreadable (colonies C15, C24 filmed on week 15). We analysed 122 videos out of 137 filmed once the surviving colonies were out of hibernation. A total of 29 colonies were alive for the 5 time points monitored after hibernation, yet 8 videos were not recorded as they had less than five workers on both weeks 45 and 47 (C3,C4,S17,S19), 5 videos were excluded during analysis after unsolvable technical issues occurred during image segmentation (C4 and C23 on week 50, C15 and S8 on week 52 and S9 on week 54) and 10 videos were lost due to disk failure during acquisition (C6, C10, C11, C17, S4, S7, S8, S10, S12,S22, all on week 50). Videos were labelled with an “S” or “C” depending on whether the queen had been treated with pathogenic conidiospores (S) or with the control treatment (C), respectively. In total, behavioural data could not be acquired from 7% (25/337) of the surviving colonies due to both biological and technical issues. We give sample sizes for each timepoint in Figures S1,S2.

*Image processing*. With the colonies established from the 2014 collection, we obtained over a thousand hours of video (more than 18TB of image data in MJPEG format), which we processed using a general multi-object tracking software written in Python (Naiser *et al.* 2018) that we

custom-adapted to detect all ants in our setup. The task of discerning the foreground (i.e. a number of similarly-sized worker ants, running alone or interacting closely with each other around the much larger ant queen) from a background of non-static brood items and detritus was achieved by training a background/foreground, pixel-based, supervised (random forest) classifier. For each frame, the output of the classifier was a probability map (i.e. a matrix, the size of the image, where an entry corresponds to the probability that a pixel belongs to the foreground), to which a blob-detection algorithm, namely MSER (Matas *et al.* 2004), was applied in order to identify groups of adjacent foreground pixels corresponding to the ant detections. The output contained a list of trajectories, defined as detections, which are assigned the same identity (i.e. matched) across consecutive frames, with the area of each detection, position, velocity, and mean-squared displacement (MSD). The MSD captures deviation of the position with respect to a reference position over time, computed as: $MSD = \{ \Sigma_i [ ( x_i - X )^2 + ( y_i - Y )^2 ] \} / n$, in which $x_i$ and $y_i$ are the coordinates of a detection in frame i, X and Y are the coordinates of its centre of mass in the trajectory considered, and n is the number of frames or length of the trajectory.

*Behavioural variables*. From the position and motion data (see Fig S2A for illustration) we derived three metrics: (1) the degree of locomotion (# workers moving / # workers), (2) the degree of foraging (# workers foraging / # workers) and (3) the degree of aggregation around the queen (# workers near the queen / # workers). The data, originally computed with a frequency of 3 Hz, were condensed to a single mean value for each filming session, which constituted the basic unit of analysis.

In detail, we considered a detection to be a single worker moving when its velocity exceeded a displacement of 1 mm between consecutive frames, its calculated MSD was at least

1.3 $mm^2$ and mean area was at most 1.8 $mm^2$. These criteria were defined based on a set of trajectories verified to contain a single worker ant (approx. 90,000 frames). For static workers, we considered them to be foraging when their position overlapped with the foraging area. Workers which gathered so tightly near the queen that individual segmentation could not be achieved, were considered in computing the degree of aggregation near the queen (Fig S2).

For each video, we input the number of ants, delineated the arena bounds and foraging area, and for a sample of 10 frames, identified spurious detections (i.e. bits of food or stains and holes in the plaster, incorrectly classified as ants) and marked the detections which contained the queen. The hand-annotated frames were used to find similar trajectories (e.g. position, size) to identify other spurious detections and to find the queen in the rest of the video, a process that was supervised with several prompts for verification. The number of ants in detections containing multiple individuals (i.e. under-segmented 'blobs') excluding that which contained the queen, was estimated using a linear model, count = round $(0.0027 \times A \times K + 0.54)$, where A is the area of the 'blob' in question and K is a quotient between the mean area of single detections in the video being processed and the video with which the model was adjusted. The model was adjusted with manual counts of n=1287 detections. We verified the estimated counts and, when necessary, adjusted blob-detection parameters to get a more accurate estimate. The accuracy of our algorithm was assessed by comparing the estimated counts to manual counts on 15-20 random frames per video. We achieved over 95% accuracy in all videos, the counts rarely being over- or underestimating worker number by more than one individual.

All code to annotate trajectories, input video metadata and to compute behavioural variables was written in Matlab. All steps which didn't require user input in the image processing and in the computation of the behavioural variables were run in the High-Performance Computing

Cluster (HPC) at IST Austria.

*Queen pathogen progression.* Using queens collected in 2018, we quantified the queens' pathogen load by quantitative real-time PCR targeting the *Metarhizium*-specific sequence of the fungal ITS2 region (as in Giehr *et al.* 2017; Stroeymeyt *et al.* 2018), to monitor pathogen progression in the queens with a focus on the course of their solitary colony founding phase and the first worker phase before hibernation, as well as once during hibernation and a last time one year after flight. To that end, entire colonies were sampled randomly and frozen at 11 time points after treatment at -80°C and stored until DNA extraction. The first time point was two hours after queen treatment (pathogen- and sham-treatment, each n=20). Between treatment and hibernation, a total of 119 queens (and their colonies) were frozen at eight fixed time points, four of them before and four after worker emergence (with our sampling regime being collection of n=12 pathogen- and n=3 sham-treated colonies per time point, except on the first week, were one less pathogen-treated colony was sampled). During hibernation, we sampled 40 pathogen- and 5 sham-treated colonies. One year after flight, the remaining 35 pathogen- and 18 sham-treated colonies were frozen (after removing some workers to assess their pathogen susceptibility, see below). Queens parasitized by a tachinid fly (n=6 pathogen- and n=1 sham-treated), plus queens (n=23 pathogen- and n=2 sham-treated) which had failed to lay any eggs after 7 weeks were excluded from analyses, as we could not exclude the possibility that their fecundity and pathogen susceptibility was otherwise affected. The colonies which died before winter (n=24 pathogen- and n=1 sham-treated) were frozen shortly after death and queen pathogen determined by qPCR. Colonies not surviving hibernation (n=32 pathogen- and n=3 sham-treated), as well as the one pathogen-treated queen dying during hibernation, were also collected and processed together, and in the same way, as the rest of the

colonies. We could not achieve qPCR quantification in two pathogen-treated queen samples, so that these colonies could only be included in the survival analysis. Final sample sizes per timepoint are detailed in Table S2.

To extract DNA, queens were transferred to a 96-well plate with sterile soft forceps and homogenized using a TissueLyser II (Qiagen), using a mixture of 1× 2.8 mm ceramic (Qiagen), 5 × 1 mm zirconia (BioSpec Products) and 110 mg of 425-600 µm glass beads (Sigma-Aldrich). Homogenization was carried out in two steps (2 x 2 min at 30 Hz). Next, DNA extraction was performed using Qiagen DNeasy 96 Blood & Tissue Kit (Qiagen, Hilden, Germany) following the manufacturer's instructions, with a final elution volume of 50 µl. The qPCRs were performed using primers specifically binding the *Metarhizium* ITS2 rRNA gene region (Met-ITS2-F: 5'-CCCTGTGGACTTGGTGTTG-3', Met-ITS2-R: 5'-GCTCCTGTTGCGAGTGTTTT-3') (Giehr *et al.* 2017). Reactions were performed in 20 µl volumes using 1× KAPA SYBR® FAST qPCR Master Mix (Kapa Biosystems), with 3 pmol of each primer (SigmaAldrich), and 2 µl of extracted DNA. The amplification program was initiated with a first step at 95°C for 5 min, followed by 40 cycles of 10 s at 95°C and 30 s at 64°C. Quantification was based on the standard curve method, using standards covering a range from 1 to $10^{-5}$ ng/µl fungal DNA (measured using a NanoDrop spectrophotometer). The standard with the lowest concentration was set as the detection threshold. Samples, as well as standards, were run in triplicates and each plate included negative controls. Specificity was confirmed by performing a melting curve analysis after each run.

*Worker pathogen susceptibility.* From colonies established from queens of the 2018 collection that survived for one year (July 2018-July 2019), we obtained worker survival data after challenge with the same pathogen (or a sham treatment). We sampled 8-25 workers from 1-year-old colonies of

15 pathogen- and 15 sham-treated queens (a total of n=174 from pathogen-treated queens and n=197 from sham-treated queens). We applied a sham treatment (n=58 and n=68, respectively from pathogen- and sham-treated queens), or challenged the workers with the same strain of *M. brunneum*, by exposing individual workers to either a high dose of approx. 30,000 infectious conidiospores/worker (by holding the worker with soft forceps and pulling them through a 0.3 μL droplet of $1\times10^8$ conidiospores/mL in 0.05% sterile TritonX that had been pipetted on a glass slide; n=56 and n=67 respectively from pathogen- and sham-treated queens), or to a very high dose of approx. 150,000 conidiospores/worker (application of 0.3 μL of $5\times10^8$ conidiospores/mL in the same way as above; n=60 and n=62 respectively from pathogen- or sham-treated queens). After treatment, workers were housed individually in a plastered petri dish (Ø=35 mm) with 10% sucrose solution, and their survival was monitored daily for 12 days. All corpses – with the exception of one sample that was lost during the process – were surface-sterilized with 70% ethanol and 5% sodium hypochlorite (as in Lacey & Brooks 1997) and placed on moist filter paper to monitor fungal outgrowth. 96% (82/85) of the corpses from a high exposure, and 97% (97/100) of the retrieved corpses from a total of 101 workers dying after a very high exposure showed *Metarhizium* outgrowth. The single ant that died in the control treatment remained without fungal outgrowth.

Statistical analyses

All analyses were performed in R-v.3.6.3 (R Core Team 2020). Unless otherwise stated, we used a generalized linear mixed modelling approach ('lme4' (Bates *et al.* 2015), 'glmmTMB' (Brooks *et al.* 2017)) in which we estimated the significance of model predictors by comparing each model to null (intercept only) and reduced models (containing all but the predictor of interest) using Likelihood Ratio (LR) tests (Bolker *et al.* 2009). We tested for interactions in the same way

(comparing to models without interaction term); when not significant, the models were refitted and run without the interaction to achieve better estimates for the main effects; when interactions were significant, the main effects were not interpreted and either separate models were run for subsets or post-hocs were performed directly on all groups to break down significant interaction effects (‘emmeans’(Lenth 2019), ‘multcomp’ (Hothorn *et al.* 2008)). We assessed model assumptions (i.e. residual normality and heterogeneity, no multicollinearity, no overdispersion), and evaluated residuals (‘DHARMa’ (Hartig 2021)) and assessed models for instability and influential observations. Effect sizes were calculated for the main models using 'performance' (Lüdecke *et al*. 2021). When multiple inferences were made, all significance values were corrected using the Benjamini-Hochberg procedure to protect against a false discovery rate of 5% (Benjamini & Hochberg 1995). All logistic regressions were implemented as GLMs with binomial error distribution and logit link function. All other graphs were made using the ‘ggplot2’ package (Wickham *et al.* 2018).

*Colony developmental trajectory.* To examine the effect of queen pathogen exposure on the brood produced across the first year of development, we built separate linear mixed-effects models for the number of larvae, pupae, and workers with queen treatment (pathogen or sham), time in weeks, and their interaction, as fixed predictors. We included random intercepts, slopes, and the interaction between these terms, to model different baselines and trajectories for each colony, as well as accounting for repeated sampling of the same colonies. For larvae and pupae, which were not present at every time point (i.e. excess zeros), the model specified was a zero-inflated model, where the probability of zero counts depended on time, and the errors had a negative-binomial distribution with log link (GLMER). In the zero-inflation part of the model, we obtained negative

estimates (i.e. as time increases the likelihood of not observing brood decreases), whereas in the conditional part of the model we obtained positive estimates (i.e. as time increases the abundance of brood becomes higher). To examine the number of workers, we specified a Gaussian error distribution with identity link (LMER). We log-transformed the number of workers, which is effectively equivalent to explicitly modeling exponential growth. The residuals of our first model revealed a pattern; a marked difference between the observations before and after winter, which was solved by including a factor that grouped data accordingly (W: before winter / after winter). This solution presented two issues: (1) potential collinearity of the grouping factor with time and (2) a three-way interaction between the predictors, which is difficult to interpret. We ran two separate simpler models, one using the data before winter and one using the data after winter, again modeling the log-transformed number of workers as a function of queen treatment, time in weeks and their interaction as fixed effects, and per colony intercept and slope as random effects to account for possible differences in colony baselines or trajectories (and their interaction in order to keep the type I error rate at the nominal level (Barr *et al.* 2013; Matuschek *et al.* 2017)). We used these separate models to extract growth rate estimates (i.e. model slopes) and tested whether growth rate before hibernation was predictive of growth after hibernation using a linear model (LM). We also compared worker number (colony size) between treatment groups (colonies of pathogen-exposed vs sham-treated queens) at the beginning of the experiment, (*ii*) just before hibernation, (iii) just after hibernation, (*iv*) and at the end of the experiment, using unpaired Wilcoxon signed-rank tests. We implemented Benjamini-Hochberg correction for all these tests (seven in total, consisting of the three linear models and four Wilcoxon signed-rank tests outlined above).

Similarly, to examine the effect of queen pathogen exposure on colony behaviour across

the first year of colony development, we built separate models (i.e. for locomotion, foraging activity, and aggregation near the queen) with queen treatment (pathogen or sham), time in weeks, and their interaction, as fixed predictors. We also included random intercepts and random slopes for each colony, and their interaction. All three behavioural variables were de-skewed by a square root transformation. For locomotion and aggregation near the queen, the model specified had a Gaussian error structure with identity link (LMER). For foraging, which was rarely detected before hibernation (i.e. excess zeros), the model specified was a zero-inflated model, where the probability of zeros (i.e. in the zero-inflated part) depended on time, the error structure was a gamma distribution with inverse link function (GLMER) and we removed the random slope term to achieve model convergence. In the zero-inflation part of the model, we obtained negative estimates (i.e. as time increases the likelihood of observing zeros decreases), whereas in the conditional part of the model we obtained positive estimates (i.e. as time increases the abundance of non-zero values increases). In addition, for the surviving colonies, we compared mean behaviours before hibernation (across four time points) to the mean behaviour after hibernation (across five time points) using paired Wilcoxon signed-rank (non-parametric) tests (results given in Fig S2).

To test whether pathogen exposure of the queen had an influence on her hibernation outcome we used a logistic regression (GLM) with a binary hibernation outcome (survive/fail) as a dependent variable and queen-treatment (pathogen vs sham) as a predictor. To investigate whether the composition of the colony at the last observed state before hibernation (week 18) influenced hibernation outcome we performed a series of logistic regressions (GLM). First, we built separate models, with either larvae, pupae or workers, as a predictor, as these variables are collinear. The number of pupae and workers, but not the number of larvae had an effect on hibernation outcome,

so we built a single model including the pupae-to-worker ratio as a single combined variable for per capita brood care investment, growth rate before hibernation, queen-treatment, and their interactions, as predictors. In a similar way, to test for the influence of colony "behavioural state" before hibernation (using week 18 data only), we built two models, one with degree of locomotion as predictor, the other with degree of aggregation near the queen (i.e. foraging was not tested as it was practically absent before hibernation). In all the above models, we included treatment and its interactions with the other predictors. The number of workers performing behaviours like locomotion, foraging and aggregation around the queen are by nature correlated to colony size. To remove any linear relationship to colony size, we used behavioural metrics that were standardised by worker number (i.e. proportion of workers showing active locomotion, foraging, or aggregation). Repeated-measures correlations with all 320 time-resolved video data (Bakdash et al. 2017) confirmed that this standardisation removed any relationship with colony size for both foraging activity ($r=0.090$, $df=271$, $P=0.135$) and locomotion ($r=-0.007$, $df=271$, $P=0.905$). The relationship between aggregation and colony size was significant, revealing a non-linear relationship, but at a very low negative r value ($r=-0.157$, $df=271$, $P=0.027$), showing that with increased colony size, the proportion of workers aggregating around the queen was over-proportionally reduced. With an absolute $r<0.2$, this can be considered a very weak relationship (Fig S3). We implemented Benjamini-Hochberg correction for all inferences drawn (twelve in total: six models looking at the effect of queen-treatment and time on composition and behaviour and six models testing whether the behavioural and compositional state of the colonies before winter predicted their hibernation outcome). To understand the relation between the behavioural metrics that predicted overwintering survival and the number of pupae present in the colony, we performed repeated measures correlation (Bakdash et al. 2017) of locomotion and aggregation near

the queen vs number of pupae, measured for each colony at different timepoints (Fig S4). Foraging was not included in the analysis as virtually no foraging activity happened before winter.

*Queen pathogen progression*. To test at which stage of the colony founding phase the queens showed detectable levels of pathogen, we modeled the probability of detecting *Metarhizium* in the pathogen-exposed queens by fitting a logistic regression (GLM) of the binary outcome of fungal detection (fungus present/absent) with the number of pupae, time of sampling in weeks, and their interaction as predictors. In addition, to estimate the extent to which the fungus was able to proliferate depending on the queen's investment into the brood, we used linear regression (LM) of fungal pathogen load (log-transformed) and the number of pupae, as a single predictor. Cox proportional-hazards (with Likelihood Ratio test, 'survival' Therneau 2021); 'survminer' Kassambara *et al.* 2021) was used to analyse queen survival depending on treatment (pathogen/sham). We also tested the effect of the pathogen on the hibernation survival of the queens, with a binomial logistic regression (GLM) of binary outcome (survive/fail) with queen-treatment as a predictor.

We also tested the effect of queen-treatment on the survival of the workers over winter, using a binomial logistic regression (GLMER) with a binomial response (workers alive vs workers dead), as a function of queen treatment and an observation-level random effect, introduced to model overdispersion. We compared final colony size (at the end of the study) between treatment groups using a Wilcoxon signed-rank test, with worker number (log-transformed) and queen treatment as a predictor. These additional tests were needed to inform our conclusions about pathogen worker susceptibility, i.e. we challenged workers taken from colonies of pathogen- and sham-treated queens, which did not differ in size ([Wilcoxon signed-rank test]: V=306, P=0.873)

and in which the proportions of workers which died in winter were not different ([GLMER] $X^2$=0.982, df=1, P=0.322).

*Worker pathogen susceptibility.* To test the susceptibility of workers originating from colonies of either pathogen- or sham-treated queens, we compared their survival after challenge with *M. brunneum*, fitting a Cox proportional hazards regression (COXPH; 'survival' (Therneau 2021), 'survminer' (Kassambara *et al.* 2021)) with a grouping variable indicating both worker treatment (high vs very high pathogen challenge, vs sham) and queen treatment (pathogen vs sham) as a main effect. We included the colony of origin as a 'cluster term' (Therneau & Grambsch 2000) in order to get a robust estimate of standard errors, given the non-dependence of observations (i.e. workers sampled from the same colony may have correlated survival probabilities). As no worker had died in the queen pathogen – worker sham treatment, this group could not be directly included in the model, but was compared to the queen sham – worker sham group by a LogRank test. The overall model significance was inferred with a Wald test and followed by Tukey contrasts ('multcomp', Hothorn *et al.* 2008) and Benjamini Hochberg adjustment.

SI TABLES

**Table S1. Experimental Schedule.** After a controlled exposure of *Lasius niger* queens to a small dose of conidiospores of the fungal pathogen *Metarhizium brunneum* or a sham treatment, we conducted a long-term cohort study (in red), where we followed the colony developmental trajectory by changes in colony behaviour (via image-analysis-based automated monitoring), composition and growth for a full year, and in particular, as they traversed the winter period (from mid November to mid April, in blue). Moreover, we examined pathogen progression inside the queens (in grey), including sampling during the solitary queen phase, before worker emergence (in yellow) and during winter. Finally, we assayed long-term worker pathogen susceptibility in workers sampled from one-year-old colonies. Dates and weeks after flight are shown; shaded dates denote observation/sampling points, non-shaded correspond to the dates of nuptial flight and collection of the queens, experimental exposure of the queens, start/end of winter conditions and the sampling of workers for the worker pathogen susceptibility assay; colony stage indicated in different colours.

| Colony developmental trajectory (2014-15) | | Queen pathogen progression (2018-19) | | |
|---|---|---|---|---|
| Date | Week after flight | Date | Week after flight | Stage |
| July 5-6 | 0 | July 3-4 | 0 | Flight / collection day |
| July 10 | 1 | July 9 | 1 | Queen exposure day |
| | | July 13 | 1 | Solitary Phase |
| | | July 27 | 3 | |
| | | Aug 10 | 5 | |
| | | Aug 24 | 7 | |
| Sep 12 | 9 | Sep 7 | 9 | First worker phase |
| Sep 29 | 12 | Sep 28 | 12 | |
| Oct 21 | 15 | Oct 19 | 15 | |
| Nov 9 | 18 | Nov 10 | 18 | |
| Nov 14 | 19 | Nov 15 | 19 | Winter |
| | | Feb 15 | 32 | |
| Apr 13 | 40 | Apr 12 | 40 | |
| May 13 | 45 | | | Ergonomic phase |
| May 31 | 47 | | | |
| June 16 | 49 | | | |
| July 1 | 52 | July 7 | 52 | >> >> |
| July 23 | 53 | July 12 | 53 | |

>> >> **Worker pathogen susceptibility** (2019)

**Table S2. Sampling scheme and sample size for queen pathogen progression.** We sampled live queens to measure their pathogen load throughout the experiment: right after queen exposure, at four timepoints during the solitary phase and four timepoints during the first worker phase before winter, once during winter, and all those which remained alive at the end of the experiment (detailed in Table S1), following the sampling scheme outlined in the methods. As pathogen infection was the focus of this experiment, and considering an expectedly higher pathogen-induced death in this group, we set up and sampled a larger number of pathogen-exposed queens. Due to several queens showing tachinid fly emergence and failing to lay eggs, final sample sizes deviated from the planned scheme to result in the numbers detailed below. Furthermore, we collected all cadavers from queens that died before hibernation (on weeks 0-19) and during hibernation (on week 40) – whilst none died after hibernation – to quantify pathogen load of all queens. Dead-sampled queens indicated by italics font.

| Stage | Week | Pathogen-treated queens | Sham-treated queens |
|---|---|---|---|
| Start of experiment | 0 | 20 | 20 |
| Solitary Phase | 1 | 11 | 3 |
| | 3 | 12 | 3 |
| | 5 | 12 | 3 |
| | 7 | 10 | 2 |
| First Worker Phase | 9 | 10 | 2 |
| | 12 | 12 | 3 |
| | 15 | 12 | 3 |
| | 18 | 10 | 3 |
| Winter | 32 | 39 | 5 |
| End of experiment | 53 | 35 | 18 |
| *Dead before hibernation* | *0-19* | *24* | *1* |
| *Dead during hibernation* | *40* | *32* | *3* |

**Table S3. Statistical results for worker development.** Analysis of variance table for a model of colony growth (worker number) and post-hoc analyses, i.e. after a three-way interaction we split the data into before winter and after winter and ran two simpler models. For the successfully-hibernating colonies, we regressed after-winter growth rate on pre-winter growth rate. We also compared numbers of workers at the beginning and end of the experiment, as well as before and after hibernation (see **Fig S1**).

<table>
<tr><th>Model specification</th><th>Dependent variable</th><th>Predictors</th><th>df</th><th>LR Test Chisq</th><th>P before BH adjustment</th><th>Sign. after BH adj.</th></tr>
<tr><td>LMER<br>colony-level random intercept<br>and time-varying slopes, Gaussian error distribution</td><td>log(workers)</td><td>overall<br><br>time ×<br>queen-trt x W (†)</td><td>7<br><br>2</td><td>326.64<br><br>22.554</td><td>< 2.2e-16<br><br>1.7e-5</td><td>***<br><br>***</td></tr>
<tr><td colspan="7">Post-hoc analyses</td></tr>
<tr><td>LMER, subset data before winter,<br>colony-level random intercept<br>and time-varying slopes, Gaussian error distribution</td><td>log(workers)</td><td>overall<br><br>time ×<br>queen-trt</td><td>3<br><br>1</td><td>80.28<br><br>7.047</td><td>< 2.2e-16<br><br>0.0079</td><td>***<br><br>*</td></tr>
<tr><td>LMER subset data after winter,<br>colony-level random intercept<br>and time-varying slopes, Gaussian error distribution</td><td>log(workers)</td><td>overall<br><br>time ×<br>queen-trt</td><td>3<br><br>1</td><td>54.92<br><br>3.152</td><td>7.1e-12<br><br>0.0758</td><td>***<br><br>·</td></tr>
<tr><td>LM</td><td>after-winter growth rate</td><td>before-winter growth rate</td><td>1</td><td>27.90</td><td>0.0062</td><td>*</td></tr>
<tr><td>Wilcoxon-signed-rank worker number comparisons</td><td>week 9<br>initial<br>V= 225, P=0.195</td><td>week 18<br>before winter<br>V= 368.5, P=0.099</td><td colspan="2">week 45<br>after winter<br>V= 111.5, P=0.512</td><td colspan="2">week 53<br>final<br>V= 85.5, P=0.407</td></tr>
<tr><td colspan="7">(†) We tested the three-way interaction between time, treatment of queens (queen-trt) and period before/after winter (W), against a model comprising each predictor and all two-way interactions.<br><br>*** P<0.001, * P<0.05, · P<0.1</td></tr>
</table>

**Table S4. Statistical results for brood development.** Analysis of variance table for the models testing the effect of queen treatment and time on the number of larvae and pupae present in the colonies (see **Fig S1**).

| Model specification | Dependent variable | Predictors | df | LR Test Chisq | P before BH adjustment | Sign. after BH adj. |
|---|---|---|---|---|---|---|
| GLMER colony-level random intercept and time-varying slopes, negative binomial error distribution, time-dependent zero-inflation(†) | larvae | overall | 2 | 18.138 | 1.15e-4 | *** |
| | | time × queen-trt | 1 | 1.716 | 0.190 | n.s. |
| | | time (§) | 1 | 18.006 | 2.20e-5 | *** |
| | | queen-trt | 1 | 0.141 | 0.707 | n.s. |
| | pupae | overall | 2 | 26.956 | 1.40e-6 | *** |
| | | time × queen-trt | 1 | 1.755 | 0.185 | n.s. |
| | | time | 1 | 26.826 | 2.22e-7 | *** |
| | | queen-trt | 1 | 0.1439 | 0.704 | n.s. |

(†) To model excess zeros, due to brood not being produced at certain time points.
(§) To achieve convergence of the reduced model and estimate the effect of the time predictor, by comparing reduced to the full model, we dropped the zero-inflation term in both models.

*** P<0.001, n.s. denotes non-significance

**Table S5. Statistical results for colony behaviour.** Analysis of variance table for the models testing the effect of queen treatment and time on worker foraging, locomotion and aggregation around the queen (see **Fig S2).**

| Model specification | Dependent variable | Predictors | df | LR Test Chisq | P before BH adjustment | Sign. after BH adj. |
|---|---|---|---|---|---|---|
| GLMER colony-level random intercept, gamma error distribution and time-dependent zero-inflation (†) | sqrt(foraging) | overall | 2 | 70.592 | 0.002 | ** |
| | | time × queen-trt | 1 | 0.451 | 0.502 | n.s. |
| | | time | 1 | 70.107 | 0.002 | ** |
| | | queen-trt (§) | 1 | 0.332 | 0.565 | n.s. |
| LMER colony-level random intercept, Gaussian error distribution | sqrt(locomotion) | overall | 2 | 15.678 | <0.001 | *** |
| | | time × queen-trt | 1 | 2.053 | 0.152 | n.s. |
| | | time | 1 | 14.698 | <0.001 | *** |
| | | queen-trt | 1 | 0.688 | 0.407 | n.s. |
| | sqrt(aggregation) | overall | 2 | 13.306 | 0.003 | ** |
| | | time × queen-trt | 1 | 1.687 | 0.194 | n.s. |
| | | time | 1 | 12.243 | <0.001 | ** |
| | | queen-trt | 1 | 1.023 | 0.312 | n.s. |

(†) To model excess zeros, due to the rarity of foraging before hibernation.
(§) To achieve convergence of the reduced model and estimate the effect of the queen-trt. predictor, by comparing reduced to the full model, we dropped the zero-inflation term in both models.

*** P<0.001, ** P<0.01, n.s. denotes non-significance

SI FIGURES

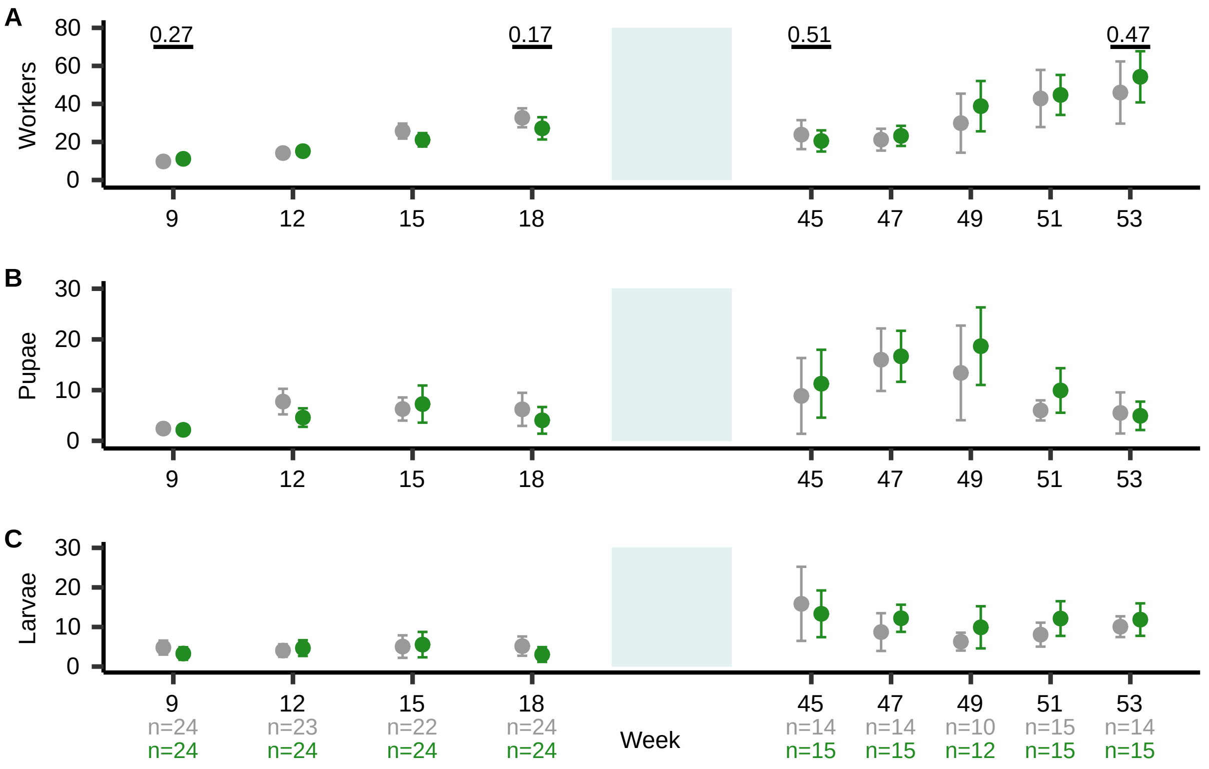


**Figure S1. Effect of queen pathogen exposure on colony composition.** Visualisation of the number of (A) workers, (B) pupae and (C) larvae in colonies founded by pathogen-treated (green) vs. sham-treated (grey) queens over the course of one year after queen flight, sampled at four time points before and five time points after winter (weeks 20-40 in pale blue). Shown are the means (across all replicates) per observation period and 95% CI. Based on a total of n=48 colonies, 24 each developing from a pathogen- or a sham-treated queen, collected in 2014; sample sizes of colonies observed specified per time point. The label n.s. refers to non-significant differences in worker numbers at the start of the experiment, before as well as after hibernation and at the end of the experiment, as assessed by unpaired Wilcoxon signed-rank tests. See Tables S3,S4 for additional statistical results.

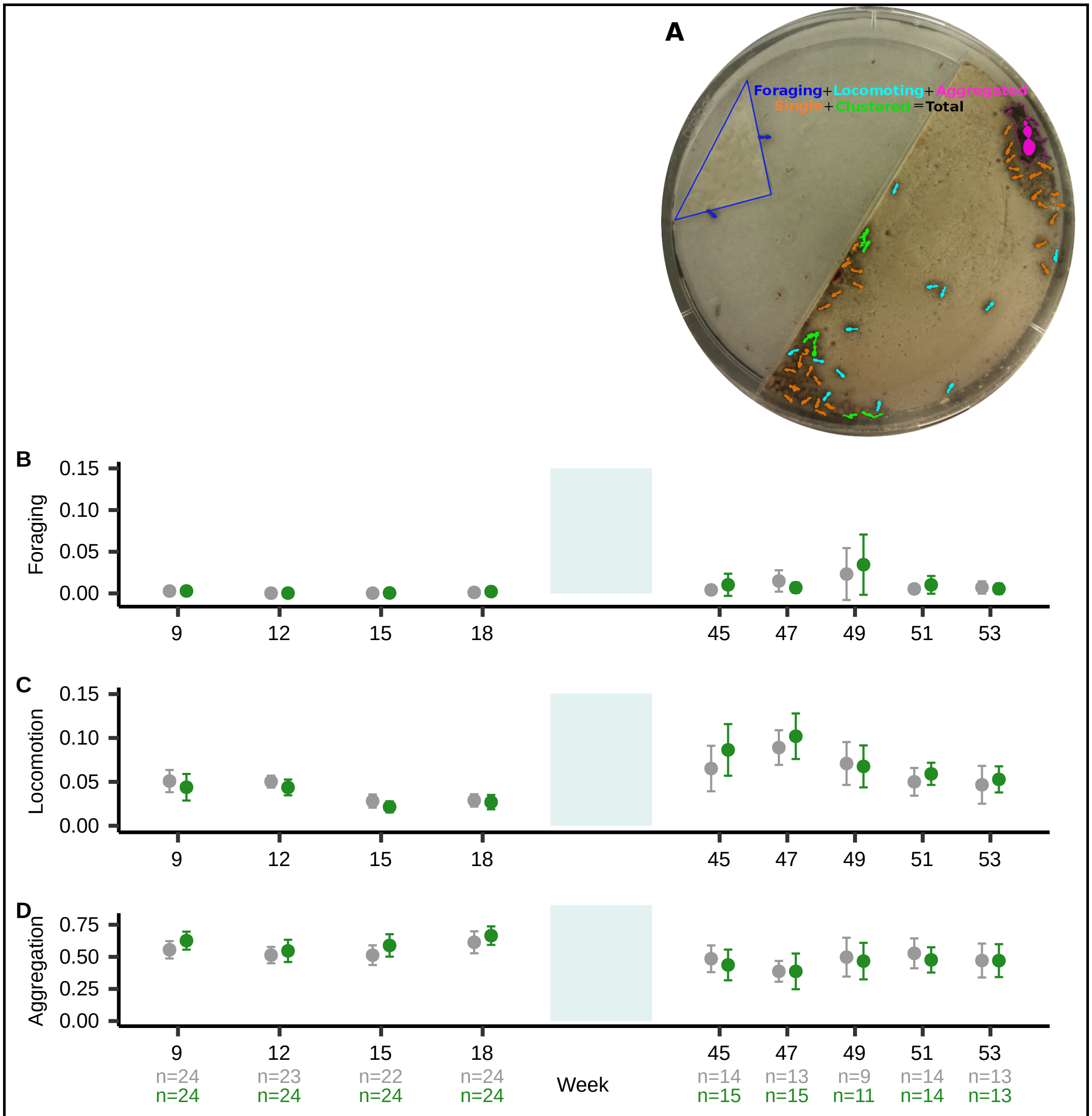


**Figure S2. Effect of queen pathogen exposure on colony behaviours.** (A) An automatized tracking algorithm was used to individually segment the ants to classify them as moving (cyan) or static (orange), or foraging (blue, above delimited area) or to identify clusters containing only workers (green) or workers aggregated near the queen (pink); example cropped frame shown.

These data were used to infer the proportion of workers foraging, moving or being aggregated around the queen (B-D). Foraging (B) was virtually only present after winter (which occurred in weeks 20-40; pale blue), but was independent of queen treatment (pathogen treatment: green; sham treatment: grey). In general, mean colony locomotion and foraging significantly increased after hibernation, whereas the aggregation near the queen decreased (Wilcoxon paired-tests among surviving colonies, aggregation: V=496; locomotion: V=30; foraging: V=17, for all tests P<0.001). Shown are the means (across all replicates) per observation period and 95% CI. Based on a total of n=48 colonies, 24 each developing from a pathogen- or a sham-treated queen, collected in 2014; sample sizes of colonies observed specified per time point. See Table S5 for statistical results.

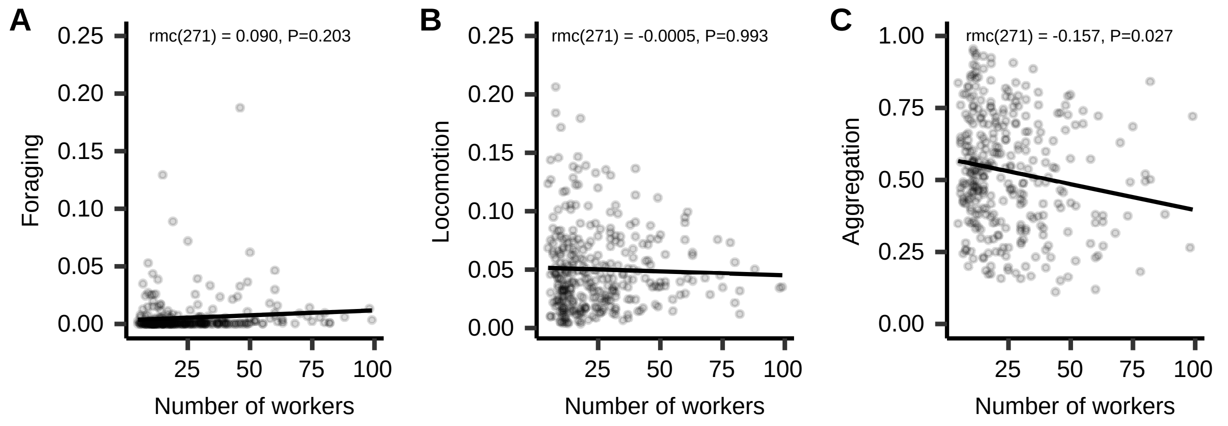


**Figure S3. Behavioural metrics are either independent or weakly-dependent on colony size.** The proportion of workers foraging (A) or being active in locomotion (B) on worker number was independent of colony size ($P>0.05$). (C) The proportion of workers aggregating around the queen was very weakly ($r=-0.157$) negatively correlated with colony size, revealing a non-linear relationship and a reduced proportion of workers in close vicinity of the queen with increasing colony size. Linear regression line depicted (black line), while repeated measures correlation performed (Bakdash *et al.* 2017) given that each colony was measured longitudinally. Based on a total of 48 colonies, 24 each developing from a pathogen- or a sham-treated queen, collected in 2014; with repeated sampling at the 9 timepoints leading to a total of 320 observations.

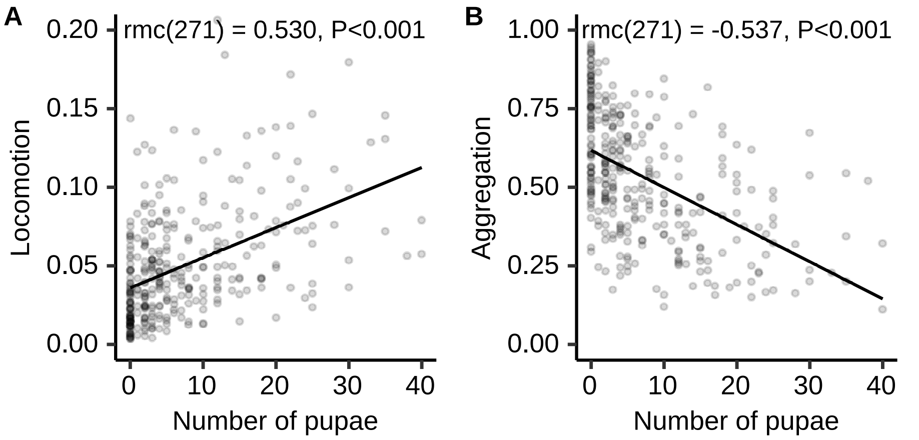


**Figure S4. Worker behaviour is dependent on pupal number.** Worker locomotion (A) increases and aggregation around the queen (B) decreases with the number of pupae in the colony. Darker dots correspond to multiple overlapping data points. Linear regression line depicted (black line), while repeated measures correlation performed (Bakdash *et al.* 2017) given that each colony was measured longitudinally. Based on a total of 48 colonies, 24 each developing from a pathogen- or a sham-treated queen, collected in 2014; with repeated sampling at the 9 timepoints leading to a total of 320 observations.

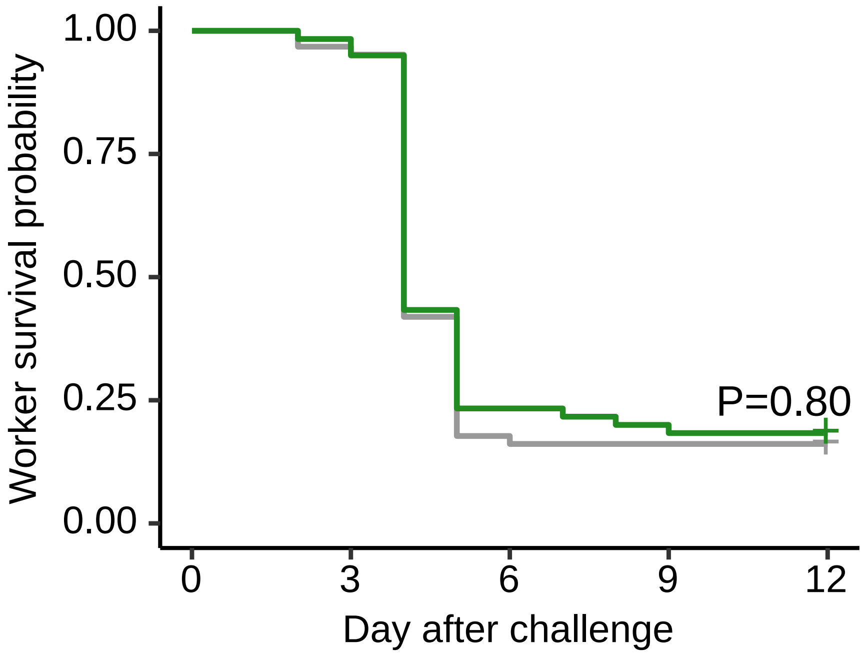


**Figure S5. Worker mortality did not depend on maternal pathogen exposure under a very high pathogen challenge.** Workers from one-year old colonies established by a pathogen-treated queen (green) did not differ in their survival from workers reared by a sham-treated queen (grey) after a very high dose (of 150,000 infectious conidiospores/worker) of the same *M. brunneum* strain that their mother had encountered a year before in a low dose. ns: non-significant difference ($P>0.05$). Based on workers from 6 pathogen- and 5 sham-treated queens, collected in 2018 (n=60 workers from pathogen- and and n=62 from sham-treated queens). 101 workers died, with fungal outgrowth monitored for 100, out of which 97 [97%] showed *Metarhizium* outgrowth.

SI REFERENCES


Bakdash, J.Z., Marusich, L.R. & Bolin, J.H. (2017). Repeated measures correlation. *Front. Psychol.,* 8, 1–13.

Barr, D.J., Levy, R., Scheepers, C. & Tily, H.J. (2013). Random effects structure for confirmatory hypothesis testing: Keep it maximal. *J. Mem. Lang.*, 68, 255–278.

Bates, D., Mächler, M., Bolker, B. & Walker, S. (2015). Fitting Linear Mixed-Effects models using lme4. *J. Stat. Softw.*, 67, 1–48.

Benjamini, Y. & Hochberg, Y. (1995). Controlling the False Discovery Rate: A practical and powerful approach to multiple testing. *J. R. Stat. Soc. Ser. B*, 57, 289–300.

Bernasconi, G. & Strassman, J.E. (1999) Cooperation among unrelated individuals: the ant foundress case. *Trends Ecol. Evol.* 14: 477-482

Bolker, B.M., Brooks, M.E., Clark, C.J., Geange, S.W., Poulsen, J.R., Stevens, M.H.H., *et al.* (2009). Generalized linear mixed models: a practical guide for ecology and evolution. *Trends Ecol. Evol.*, 24, 127–135.

Brooks, M.E., Kristensen, K., van Benthem, K.J., Magnusson, A., Berg, C.W., Nielsen, A., *et al.* (2017). glmmTMB balances speed and flexibility among packages for zero-inflated Generalized inear Mixed Modeling. *R J.*, 9, 378–400.

Giehr, J., Grasse, A. V, Cremer, S., Heinze, J. & Schrempf, A. (2017). Ant queens increase their reproductive efforts after pathogen infection. *R. Soc. Open Sci.*, 4, 1–8.

Hakala, S.M., Seppä, P. & Helanterä, H. (2019). Evolution of dispersal in ants (Hymenoptera: Formicidae): a review on the dispersal strategies of sessile superorganisms. *Myrmecol. News*, 29, 35–55.

Hart, A., Hesselberg, T., Nesbit, R. & Goodenough, A. (2017). The spatial distribution and environmental triggers of ant mating flights: Using citizen-science data to reveal national

patterns. *Ecography*, 41, 10.1111/ecog.03140.

Hartig, F. (2021). DHARMa: Residual Diagnostics for Hierarchical (Multi-Level / Mixed) Regression Models. R package version 0.4.1.

Hothorn, T., Bretz, F. & Westfall, P. (2008). Simultaneous Inference in General Parametric Models. *Biometrical Journal,* 50, 346–363

Kassambara, A., Kosinski M. & Biecek, P. (2021). survminer: Drawing survival curves using ‘ggplot2’. R package version 0.4.9.

Konrad, M., Vyleta, M.L., Theis, F.J., Stock, M., Tragust, S., Klatt, M., *et al.* (2012). Social transfer of pathogenic fungus promotes active immunisation in ant colonies. *PLOS Biol.,* 10, 1–15.

Lacey, L.A. & Brooks, W.M. (1997). *Manual of Techniques in Insect Pathology, Chapter I.* Academic Press.

Lenth, R.V. (2021). emmeans: Estimated Marginal Means, aka Least-Squares Means. R package version 1.6.1.

Lüdecke D., Ben-Shachar, M., Patil, I., Waggoner, P., Makowski, D. (2021). performance: An R Package for Assessment, Comparison and Testing of Statistical Models. *J. Open Source Software,* 6(60), 3139.

Matas, J., Chum, O., Urban, M. & Pajdla, T. (2004). Robust wide-baseline stereo from maximally stable extremal regions. *Image Vis. Comput.*, 22, 761–767.

Matuschek, H., Kliegl, R., Vasishth, S., Baayen, H. & Bates, D. (2017). Balancing Type I error and power in linear mixed models. *J. Mem. Lang.*, 94, 305–315.

Naiser, F., Šmíd, M. & Matas, J. (2018). Tracking and Re-Identification System for Multiple Laboratory Animals. *Vis. Obs. Anal. Vertebr. Insect Behav. Work. ICPR.*

Pull, C.D. & Cremer, S. (2017). Co-founding ant queens prevent disease by performing prophylactic undertaking behaviour. *BMC Evol. Biol.*, 17.

Pull, C.D., Hughes, W.O.H. & Brown, M.J.F. (2013). Tolerating an infection: an indirect benefit of co-founding queen associations in the ant *Lasius niger*. *Naturwissenschaften*, 100, 1125–1136.

R Core Team. (2020). R: A Language and environment for statistical computing. R Foundation for Statistical Computing, Vienna, Austria.

Stroeymeyt, N., Grasse, A. V., Crespi, A., Mersch, D.P., Cremer, S. & Keller, L. (2018). Social network plasticity decreases disease transmission in a eusocial insect. *Science,* 362, 941–945.

Therneau T.M. & Grambsch, P.M. (2000). *Modeling survival data: extending the Cox Model*. Springer, New York.

Therneau, T.M. (2021). A Package for Survival Analysis in R_. R package version 3.2-11.

Wickham, H., Chang, W., Henry, L., Pedersen, T.L., Takahashi, K., Wilke, C., *et al.* (2018). ggplot2: create elegant data visualisations using the grammar of graphics. R package version 3.3.3.
